# Sb_2_S_3_@PPy Coaxial Nanorods: A Versatile and Robust Host Material for Reversible Storage of Alkali Metal Ions

**DOI:** 10.3390/nano9040560

**Published:** 2019-04-06

**Authors:** Yang Shi, Feng Li, Yi Zhang, Liang He, Qing Ai, Wen Luo

**Affiliations:** 1 State Key Laboratory of Advanced Technology for Materials Synthesis and Processing, Wuhan University of Technology, Wuhan 430070, China; shiyang@whut.edu.cn (Y.S.); 211779@whut.edu.cn (Y.Z.); hel@whut.edu.cn (L.H.); 2 Hefei National Laboratory for Physical Sciences at the Microscale, University of Science and Technology of China, Hefei 230000, China; fengli96@mail.ustc.edu.cn; 3 School of Materials Science and Engineering, Shandong University, 17923 Jingshi Road, Jinan 250061, China; qai@mail.sdu.edu.cn; 4 Department of Physics, School of Science, Wuhan University of Technology, Wuhan 430070, China

**Keywords:** chalcogenide, one-dimensional nanomaterials, Sb_2_S_3_@PPy, alkali metal ions batteries, anode, energy storage mechanism

## Abstract

Chalcogenides have attracted great attention as functional materials in optics, electronics, and energy-related applications due to their typical semiconductor properties. Among those chalcogenides, Sb_2_S_3_ holds great promise in energy storage field, especially as an anode material for alkali metal (Li, Na, and K) batteries. In this work, a one-dimensional coaxial Sb_2_S_3_@PPy is investigated as a versatile and robust anode in three kinds of alkali metal batteries for the first time, and the energy storage mechanism of these batteries is systematically discussed. As an anode material for sodium ion batteries (SIBs) and potassium ion batteries (KIBs), Sb_2_S_3_@PPy exhibits high reversible capacity and impressive cycle lifespan. Sb_2_S_3_@PPy anode demonstrates an adsorption behavior that has a significant influence on its sodium storage behavior, providing a universal model for studying the application of chalcogenide compounds.

## 1. Introduction

Chalcogenides are important inorganic materials with excellent optical and electrical properties. Thus, they are widely applied in photocatalysis [1], supercapacitors [2], solar cells [3], and batteries [4,5,6]. As an important kind of chalcogenides, metal sulfide is one of the research hotspots in recent years. For example, Zhao et al. [7] proposed that n-type PbS achieved a high dimensionless figure of merit (ZT) value of 1.1 at 923 K. Efren et al. [8] reported two-dimensional (2D) superconductivity of atomically thin 2H-TaS_2_. In recent years, chalcogenides have received extensive attention in the energy storage field owing to the ultra-high theoretical specific capacity. For example, Hu et al. [9] first reported FeS_2_ as an anode for sodium ion batteries (SIBs), and the assembled half cell showed excellent cycling performance (90% after 20,000 cycles) and rate performance (170 mAh g^−1^ at 20 A g^−1^).

Recently, Sb_2_S_3_ emerged as a versatile and promising functional material widely applied in various fields. For example, Chang et al. [3] used Sb_2_S_3_ as an absorbing semiconductor in solar cells, owing to its high absorption coefficient (1.8 × 10^5^ cm^−1^ at 450 nm) and optical band gap (E_g_ = 1.7 eV). Zhang et al. [10] incorporated Sb_2_S_3_ onto WO_3_, and its photoelectroncatalytic activity under visible-light illumination was improved. Among them, the application in battery field is particularly attractive, as evidenced by an increasing number of research works [11,12,13], because the high theoretical capacity (946 mAh g^−1^) of Sb_2_S_3_ is much higher than that of commercial graphite. For example, Xiong et al. [11] prepared S-doped graphene sheets (SGS)-supported Sb_2_S_3_ as SIBs anodes, which delivered high capacity, good rate performance, and excellent cyclic stability. The calculation result of density functional theory (DFT) showed that the SGS had a stronger affinity for Sb_2_S_3_ and intermediate products, demonstrating the more stable structure of the SGS supported Sb_2_S_3_, which remarkably strengthens its cyclic stability. With respect to the development of next-generation high-performance batteries, potassium ion batteries (KIBs) are gradually attracting much interest [14,15,16]. Compared with lithium, potassium resource is more abundant [17,18]. In addition, the redox potential of K/K^+^ (−2.93 V versus standard hydrogen electrode) is lower than that of Na/Na^+^ (−2.71 V), implying that KIBs have a high voltage plateau and high energy density. Liu et al. [19] firstly investigated Sb_2_S_3_ for KIBs anode and synthesized a few-layered Sb_2_S_3_/carbon sheets composite. The synthesized composite showed a high reversible capacity (404 mAh g^−1^ after 200 cycles) and good rate capability. However, there are few systematic studies on the electrochemical behavior of Sb_2_S_3_ in alkali metal based batteries, and differences between lithium, sodium, and potassium storage behavior are still unknown. Moreover, even though Sb_2_S_3_ has a high theoretical capacity, its cyclability and rate performance still need to be improved.

Generally, nanostructured materials can shorten ion diffusion paths and improve the conductivity [20,21], and conductive coating can enhance its cycle stability [22]. Some work also demonstrates that polymer materials with abundant functional groups on the surface enhance the ion adsorption capability of the material, positively affecting its electrochemical performance in supercapacitor and battery applications [23]. Herein, we synthesized the Sb_2_S_3_ nanorods through a facile hydrothermal method [24]. Then, in order to improve the cycle stability of Sb_2_S_3_, a low-cost polypyrrole (PPy) layer was coated on the surface of Sb_2_S_3_ nanorods by a room temperature stirring method, and Sb_2_S_3_@PPy with a one-dimensional coaxial structure was obtained. Afterward, we systematically explored the electrochemical performances of Sb_2_S_3_@PPy nanorods as anode materials of alkali metal batteries, including lithium ion batteries (LIBs), SIBs, and KIBs. Through efficient coating, the Sb_2_S_3_@PPy nanorods as LIBs and SIBs anodes had excellent cycle stability and rate performance. The electrochemical performance of the synthesized Sb_2_S_3_@PPy coaxial nanorods was higher than those reported of sulfide-polymer anodes (detailed results are discussed in the following section). More importantly, it was found that the Sb_2_S_3_@PPy nanorods also showed excellent electrochemical performance in KIBs. A universal model based on alkali metal batteries was developed, showing wide applications in alkali metal ions storage. In the study of lithium and sodium storage, it was found that their energy storage behaviors were different, and the adsorption behavior had a significant influence during the sodium storage process. Our work provides a research model of versatile material for the future research of chalcogenides applied in alkali metal rechargeable batteries.

## 2. Materials and Methods

### 2.1. Synthesis of Sb_2_S_3_ Nanorods

First, 1.92 g Na_2_S·9H_2_O (Alfa Aesar Chemicals Co., Ltd, Shanghai, China), 0.969 g C_3_H_7_NO_2_S (Alfa Aesar Chemicals Co., Ltd, Shanghai, China), and 0.912 g SbCl_3_ (Alfa Aesar Chemicals Co., Ltd, Shanghai, China) were added into 80 mL distilled water sequentially. After stirring for 3 h, the mixed solution was transferred into a 100 mL Teflon-lined autoclave and heated at 180 °C for 12 h. After the solution was cooled down to the ambient temperature, the precipitate was obtained by centrifugation and washed with distilled water and alcohol several times. The Sb_2_S_3_ powders were obtained after being dried under vacuum at 80 °C for 8 h [24].

### 2.2. Synthesis of Sb_2_S_3_@PPy Coaxial Nanorods

The synthesized Sb_2_S_3_ nanorods were further coated by PPy through a solution reaction [24]. Then, 4 mg sodium dodecyl sulfate and 80 mg prepared Sb_2_S_3_ nanorods were added into 40 mL deionized (DI) water followed by dispersion using sonication for 0.5 h. After mild stirring for 1 h, 21 μl pyrrole (Alfa Aesar Chemicals Co., Ltd, Shanghai, China) monomer was added into the solution, and after vigorously stirring for 1 h, 4 mL with 0.1 mol L^−1^ (NH_4_)_2_S_2_O_8_ (Alfa Aesar Chemicals Co., Ltd, Shanghai, China) as the oxidant was added to the above solution drop-wise. After stirring for another 4 h, the product was collected by centrifuging and washing, followed by a freeze-drying process overnight.

### 2.3. Materials Characterization

The crystalline structure of the samples was measured by a Bruker D8 Advance X-ray diffractometer (Bruker, Karlsruhe, Germany) using Cu Kα radiation. Raman spectra of the samples were obtained on a Renishaw in Via Raman microscope (Horiba, Tokyo, Japan) with a 632.8 nm He-Ne laser. SEM images and energy dispersive spectroscopy (EDS) results were obtained using a JEOL7100F SEM/EDS microscope (JEOL, Tokyo, Japan). TEM and high resolution TEM (HRTEM) images were collected on a JEM-2100F scanning transmission electron microscopy (STEM)/EDS microscope (JEOL, Tokyo, Japan). FT-IR spectra were collected on a 60-SXB IR spectrometer (Nicolet, Madison, Wisconsin, USA).

### 2.4. Electrochemical Performance

The electrochemical behaviors of samples were characterized through assembly of 2016 coin cells in the argon-filled glove box. The pure Li, Na, or K discs were used as the counter electrode, and the working electrode consisted of Sb_2_S_3_@PPy nanorods, ketjen black, and carboxyl methyl cellulose with a weight ratio of 7:2:1 on a copper foil. The electrolyte was 1 M LiPF_6_ in ethylene carbon (EC)/dimethyl carbonate (DMC)/ethyl methyl carbonate (EMC) (1:1:1 by volume) for LIBs, 1 M NaClO_4_ in EC/DMC (1:1 by volume) with 5% fluorinated ethylene carbonate for SIBs, and 0.8 M KPF_6_ in EC/DEC (diethyl carbonate) with a volume ratio of 1:1 for KIBs. The galvanostatic charge-discharge tests were conducted through a multichannel battery testing system (LAND CT2001A), and cyclic voltammetry (CV) curves were collected using an electrochemical workstation (Autolab PGSTAT 302).

## 3. Results and Discussion

### 3.1. Materials Synthesis and Characterization

**Figure 1:**
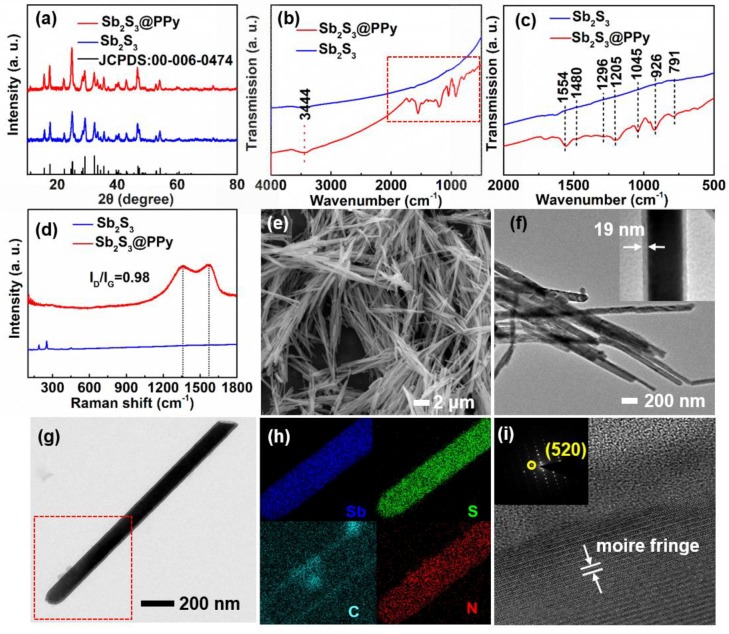
(**a**) XRD patterns of Sb_2_S_3_ and Sb_2_S_3_@PPy nanorods. (**b**) FT-IR spectra of Sb_2_S_3_ and Sb_2_S_3_@PPy nanorods (wavenumber: 500–4000 cm^−1^). (**c**) FT-IR spectra of Sb_2_S_3_ and Sb_2_S_3_@PPy nanorods (wavenumber: 500–2000 cm^−1^). (**d**) Raman spectra of Sb_2_S_3_ and Sb_2_S_3_@PPy nanorods. (**e**) SEM images of Sb_2_S_3_@PPy nanorods. (**f**,**g**) TEM images of Sb_2_S_3_@PPy nanorods. (**h**) Element mapping images of Sb_2_S_3_@PPy nanorods. (**i**) High resolution TEM (HRTEM) image and selected area electron diffraction (SAED) pattern of Sb_2_S_3_@PPy nanorods.

The Sb_2_S_3_@PPy coaxial nanorods were prepared by two steps. First, Sb_2_S_3_ nanorods were prepared using a hydrothermal method. Surfactant, antimony, and sulphur sources were added into DI water. After stirring, the mixture was transferred into an autoclave for a hydrothermal reaction. Then, as the template, Sb_2_S_3_ nanorods were coated with a homogeneous PPy layer through a solution reaction. Figure 1a displays the XRD patterns of bare Sb_2_S_3_ and Sb_2_S_3_@PPy. All diffraction peaks of the two samples were well indexed to the Sb_2_S_3_ (JCPDS (Joint Committee on Powder Diffraction Standards): 00-006-047), demonstrating the pure antimonite phase of Sb_2_S_3_ and Sb_2_S_3_@PPy nanorods, suggesting PPy coating did not affect the crystal structure of Sb_2_S_3_. Figure 1b,c shows FT-IR spectra of Sb_2_S_3_ and Sb_2_S_3_@PPy. Before coating, no distinctive FT-IR adsorption peak could be observed for the Sb_2_S_3_ sample. With regard to Sb_2_S_3_@PPy, the peaks at 1554 and 1480 cm^−1^ corresponded to the typical ring vibrations of PPy. The peak at 3444 cm^−1^ corresponded to the vibration with the N-H bond. The C-N stretching vibrations of the benzoid was depicted by the peak at 1296 cm^−1^. The peaks at 1205 and 1045 cm^−1^ corresponded to the breathing vibration of the PPy ring and the C-H deformation, respectively. The C-H wagging was proved by the band at 965 and 791 cm^−1^ [25]. All of these results confirmed the PPy was coated on the Sb_2_S_3_ nanorods. Raman spectrum of Sb_2_S_3_ indicated their carbon characteristic. The bands at 1365 and 1580 cm^−1^ corresponded to the D-band (disordered carbon) and G-band (graphitic carbon) [26], respectively. The I_D_/I_G_ ratio of Sb_2_S_3_@PPy nanorods (≈0.98) represented a relatively high graphitization degree of the sample (Figure 1d). Morphology characteristics of the samples were demonstrated in SEM and TEM images. As shown in Figure 1e, the Sb_2_S_3_@PPy sample showed a uniform nanorod morphology. The length of the nanorods ranged from 3 to 15 μm, and the diameter ranged from 100 to 200 nm. Figure 1f, g revealed the nanostructure of the sample, and the Sb_2_S_3_ nanorods were coated with a 19 nm thick PPy layer. Furthermore, the elemental mapping (Figure 1h) results showed that Sb, S, C, and N elements were evenly distributed within the sample, further illustrating that PPy was uniformly coated on the Sb_2_S_3_ nanorods. Figure 1g shows the moire fringe of the Sb_2_S_3_@PPy nanorods, indicating that the Sb_2_S_3_@PPy nanorods had good dispersion and were not agglomerated. Furthermore, the selected area electron diffraction (SAED) pattern of the Sb_2_S_3_@PPy nanorods indicated the single-crystal characteristic. All characterization results showed that the synthesized Sb_2_S_3_@PPy nanorods had uniform morphology and good dispersion.

### 3.2. Lithium Storage Performances

**Figure 2:**
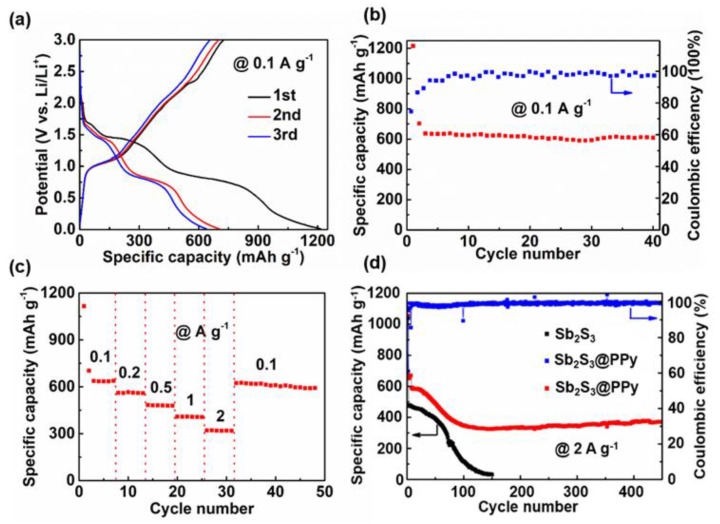
Electrochemical performances of Sb_2_S_3_@PPy or Sb_2_S_3_ as an anode in lithium ion batteries (LIBs). (**a**) Galvanostatic charge and discharge curves of Sb_2_S_3_@PPy at 0.1 A g^−1^ for the first three cycles. (**b**) Cycling stability and corresponding Coulombic efficiency of Sb_2_S_3_@PPy at 0.1 A g^−1^. (**c**) Rate capability of Sb_2_S_3_@PPy from 0.1 to 2 A g^−1^. (**d**) Cycling stability of Sb_2_S_3_@PPy and Sb_2_S_3_ at 2 A g^−1^, and corresponding Coulombic efficiency of Sb_2_S_3_@PPy at 2 A g^−1^.

The lithium storage performances of Sb_2_S_3_ and Sb_2_S_3_@PPy nanorods were evaluated in half cells. Figure 2a exhibits the charge and discharge voltage profiles of Sb_2_S_3_@PPy at 0.1 A g^−1^. The initial discharge and charge capacities of the working electrode were 1215.7 and 727.4 mAh g^−1^, respectively. Additionally, the initial curve was characterized by three discharge platforms at 1.7, 1.3, and 0.7 V, respectively, consistent with reported results [27]. As shown in Figure 2b, Sb_2_S_3_@PPy nanorods exhibited a competitively high capacity and good stability after cycling. The capacity remained at 608 mAh g^−1^ after 40 cycles (95% capacity retention). The Coulombic efficiency of the initial cycle was 59.8%, which was a result of the formation of solid electrolyte interphase (SEI) film. Then, the Coulombic efficiency gradually increased in the following six cycles, and then it remained around 100%. This result was caused by the fact that intermediate polysulfides created during the electrochemical reactions tended to dissolve in the electrolyte according to Equation (1). Equation (2) displays the alloying process.

Sb_2_S_3_ + 6Li^+^ + 6e^−^ → 2Sb + 3Li_2_S (1)

2Sb + 6Li^+^ + 6e^−^ → 2Li_3_Sb (2)

Furthermore, the capacity of Sb_2_S_3_@PPy nanorods remained 597 mAh g^−1^ after 40 cycles (97.8% capacity retention). Figure 2c displays the rate performance of Sb_2_S_3_@PPy nanorods. With the current density increasing from 0.1 to 2 A g^−1^, the capacities were 637.8, 560, 480, 410, and 310 mAh g^−1^, respectively. When the current density reverted back to 0.1 A g^−1^, the capacity quickly recovers to 610 mAh g^−1^, verifying good structural stability and electrochemical reversibility of Sb_2_S_3_@PPy. Figure 2d displays the long cycling performance of the samples. The capacity of Sb_2_S_3_@PPy remained at 390 mAh g^−1^ at 2 A g^−1^ after 400 cycles, while the capacity of the Sb_2_S_3_ nanorods almost attenuated to 0 mAh g^−1^ after 150 cycles. It has been reported that under high current density, the decay in capacity is due to the decrease in crystallinity caused by the aggregation of the material [28]. However, in our case, due to the effective PPy coating, even though a moderate capacity decrease was observed in the beginning 100 cycles, Sb_2_S_3_@PPy still maintained a relatively high capacity. In contrast, the capacity of Sb_2_S_3_ without PPy coating faded to almost 0 mAh g^−1^ after 150 cycles, demonstrating that the cycling performance of Sb_2_S_3_ nanorods was enhanced obviously by PPy coating.

### 3.3. Sodium Storage Performance

**Figure 3:**
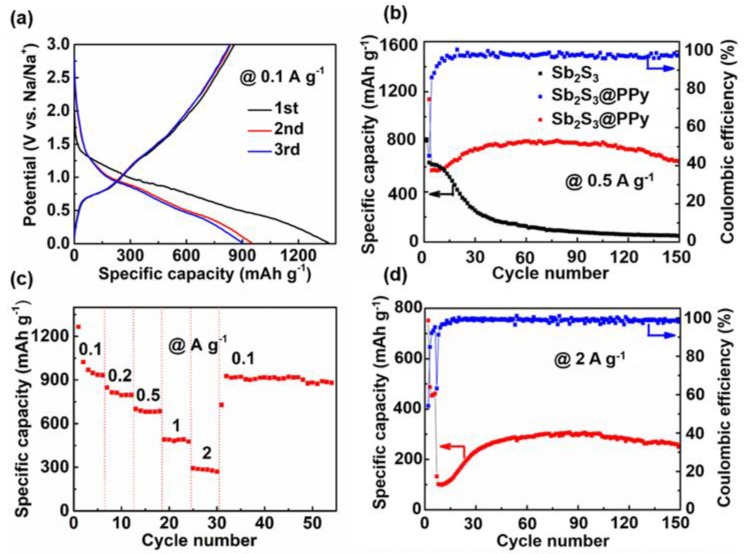
Electrochemical performances of Sb_2_S_3_@PPy or Sb_2_S_3_ as an anode in sodium ion batteries (SIBs). (**a**) Galvanostatic charge and discharge curves of Sb_2_S_3_@PPy at 0.1 A g^−1^ for the first three cycles. (**b**) Cycling stability of Sb_2_S_3_@PPy and Sb_2_S_3_ at 0.5 A g^−1^, and corresponding Coulombic efficiency of Sb_2_S_3_@PPy at 0.5 A g^−1^. (**c**) Rate capability of Sb_2_S_3_@PPy from 0.1 to 2 A g^−1^. (**d**) Cycling stability and corresponding Coulombic efficiency of Sb_2_S_3_@PPy at 2 A g−1.

The sodium storage behavior of Sb_2_S_3_@PPy was studied using the same method. Figure 3a displays the charge and discharge profiles of Sb_2_S_3_@PPy nanorods for first three cycles at 0.1 A g^−1^. Its initial discharge and charge capacities were 1350 and 860 mAh g^−1^, respectively. Compared with LIBs, the charge-discharge curve had no obvious voltage platform, indicating that it was more similar as a capacitor during charge and discharge processes. Figure 3b displays the cycling performance of the samples. The Sb_2_S_3_@PPy nanorods had an initial reversible capacity of 580 mAh g^−1^ at 0.5 A g^−1^, which remained at 632 mAh g^−1^ after 150 cycles, and the Coulombic efficiency was maintained at about 100%. Meanwhile, the Sb_2_S_3_ nanorods had only 30% capacity retention after 30 cycles. During charge and discharge processes, there was a gradual increase in the capacity from the first cycle to the thirtieth cycle. The originally inserted Na ions were extracted after 30 cycles, contributing to the increase in capacity [29]. Figure 3c shows the rate capability of Sb_2_S_3_@PPy nanorods, and the average capacity of the initial six cycles was 940 mAh g^−1^ at 0.1 A g^−1^. As the current densities increased to 0.2, 0.5, 1, and 2 A g^−1^, the average capacities were 807, 690, 490, and 290 mAh g−1, respectively. When the current density reverted back to 0.1 A g^−1^ after 30 cycles, its capacity recovered to 920 mAh g^−1^. Figure 3d shows the long-term cycling performance of Sb_2_S_3_@PPy at 2 A g^−1^. During the cycling process, the capacity also suffered a slowly rising trend, which was similar as that at low current densities. After 150 cycles, the capacity retained at 275 mAh g^−1^, equivalent to the initial reversible capacity.

The Sb_2_S_3_@PPy had a good sodium storage performance; specifically, the reversible capacity and the rate performance were higher than those reported of MWNTs@Sb_2_S_3_@PPy [30] and flowerlike Sb_2_S_3_@PPy microspheres [31]. Interestingly, the storage behavior was different from lithium storage. First, capacity suffered an increase during the first 30 cycles and then remained in a stable state. Second, the electrode did not present an obvious charge and discharge platform, which did not resemble the electrochemical behavior in LIBs, and its energy storage mechanism was more similar to a capacitor than to LIBs. The reason for these differences remains unclear. It was assumed to be related to the solvation of sodium ion electrolyte with the surface charge groups of electrode material.

### 3.4. Potassium Storage Performance

Until now, only few works had studied the potassium storage performance of Sb_2_S_3_ [29]. In our work, KIBs were assembled through the same method. Sb_2_S_3_@PPy nanorods were paired with a metallic K foil.

**Figure 4:**
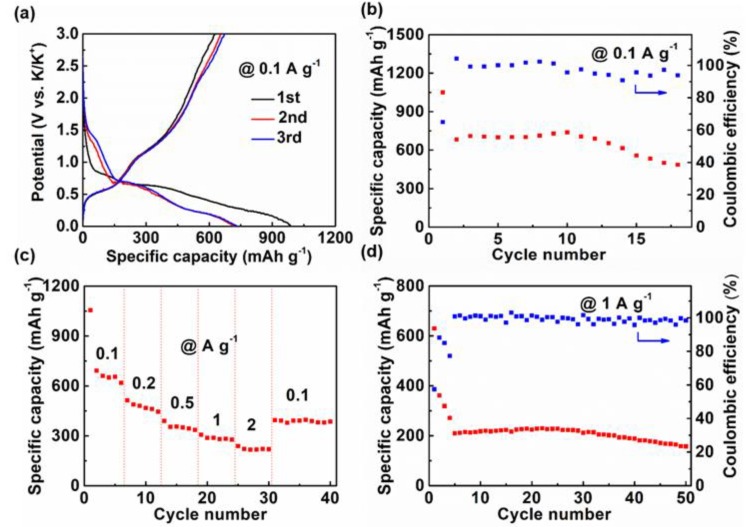
Electrochemical performances of Sb_2_S_3_@PPy as an anode in potassium ion batteries (KIBs). (**a**) Galvanostatic charge and discharge curves at 0.1 A g^−1^ for the first three cycles. (**b**) Cycling stability and corresponding Coulombic efficiency at 0.1 A g^−1^. (**c**) Rate capability from 0.1 to 2 A g^−1^. (**d**) Cycling stability and corresponding Coulombic efficiency at 1 A g^−1^.

The constant current charge and discharge curves of KIBs are shown in Figure 4a. During the discharge process, there was an inclined platform around 0.7 and 0.3 V, respectively, and in the charge process, there was also a platform at 0.6 and 1.3 V, respectively, corresponding to the conversion and alloying reaction [17]. The initial discharge and charge capacities were 986.2 and 628.1 mAh g^−1^, respectively, and the corresponding Coulombic efficiency was 63.7%. Figure 4b delivers the cycle performance of Sb_2_S_3_@PPy nanorods. The reversible capacity remained at 487 mAh g^−1^ after 18 cycles, and a Coulombic efficiency near 100% during charge and discharge processes was obtained. Figure 4c shows the rate capacity of Sb_2_S_3_@PPy nanorods. The reversible capacity was 690 mAh g−1 at 0.1 A g^−1^, which remained at 220 mAh g^−1^ when the current density increased to 2 A g^−1^. The long cycle performance at 1 A g^−1^ is displayed in Figure 4d. The capacity of Sb_2_S_3_@PPy nanorods remained at 157 mAh g^−1^ after 50 cycles.

Sb_2_S_3_@PPy coaxial nanorods had a great electrochemical performance when applied in KIBs. The energy storage mechanism was a conversion reaction and an alloying reaction [17]. Unlike the sodium storage process, there was a voltage platform in the charge and discharge curve, but it was not as obvious as that in LIBs. Its initial reversible capacity was up to 700 mAh g^−1^ at 0.1 A g^−1^, higher than those of reported Sb_2_S_3_ materials.

At last, the performances of SIBs and KIBs in our work were compared with the reported works. As shown in Table 1, the synthesized Sb_2_S_3_@PPy coaxial nanorods had a competitive electrochemical performance in SIBs and KIBs. Especially in SIBs, its reversible capacity was much higher than those reported of the sulfide-polymer anodes.

### 3.5. Alkali Metal Ions Storage Mechanism

**Figure 5:**
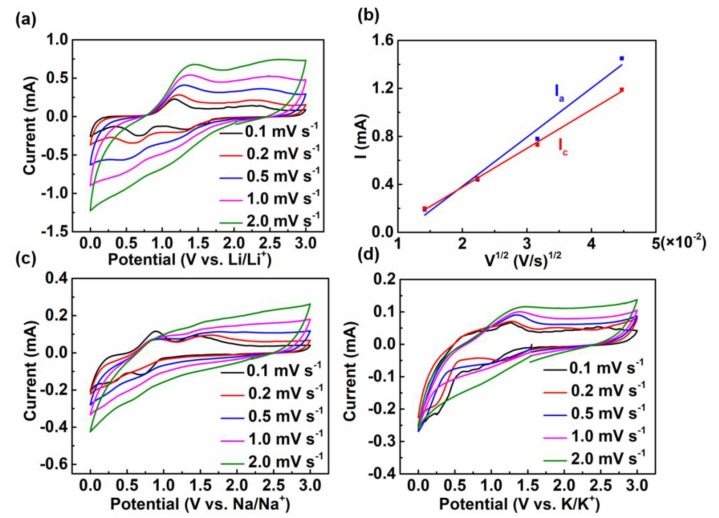
(**a**) Cyclic voltammetry (CV) curves of LIBs at different scan rates. (**b**) Cathodic and anodic peak currents versus the square root of scan rate, data are obtained from part (**a**). (**c**) CV curves of SIBs at different scan rates. (**d**) CV curves of KIBs at different scan rates.

In order to investigate the charge storage mechanism, the CV curves at various scan rates of LIBs, SIBs, and KIBs were recorded. Figure 5a exhibits the CV curves of LIBs at various scan rates from 0.1 to 2 mV s^−1^. In the CV curves, an obvious anodic peak at 1.24 V and two cathodic peaks at 0.75 and 1.26 V illustrated the lithiation and delithiation processes of Sb_2_S_3_@PPy nanorods, respectively. These peaks also corresponded to the voltage platform in the charge and discharge curve. When the peak current was plotted with respect to the square root of the scanning rate (v^1/2^), a linear relationship was obtained (Figure 5b). The result obviously proved that the Li^+^ storage in Sb_2_S_3_@PPy nanorods proceeded via a diffusion-limited faradaic mechanism instead of a surface-mediated capacitive mechanism [28]. The CV curves of SIBs are displayed in Figure 5c; at 0.1 mV s^−1^, there was a significant anodic peak at 0.8 V. Moreover, as the scan rates increased, the CV curve approached a rectangular similar to the capacitor, which was consistent with the charge and discharge profiles. Figure 5d delivers the CV curves of KIBs at different scan rates. There was an obvious anodic peak at 1.3 V and an unobvious peak around 0.8 V in the CV curve, corresponding to the alloying reaction and the conversion process.

By comparing the CV curves of LIBs, SIBs, KIBs, it was found that all batteries had obvious redox peaks at low scan rates during charge and discharge. However, as the scan rates increased to 2 mV s^−1^, SIBs and KIBs did not have obvious redox peaks. The storage behavior in SIBs and KIBs was similar to the capacitor under high scan rates. In order to further study the charge storage mechanisms in LIBs, SIBs, and KIBs, we calculated the surface diffusion coefficients of three ions (Li^+^, Na^+^, and K^+^) in alkali metal batteries using the equation as follows [24]:

I_p_ = 2.69 × 10^5^ACD^1/2^n^3/2^ν^1/2,^

in which I_p_ is the peak current, A means the surface area of active materials in anode, C is the ions concentration, D is the apparent alkali metal ions diffusion coefficient, n represents the number of electrons transferred per molecule during the electrochemical reaction, and v means scan rate. As shown in Table 2, the diffusion coefficient of Li^+^ was higher than those of Na^+^ and K^+^. The reasons for this result were explained as follows. First, the Li^+^ diffusion coefficient was the highest, which was because of the larger radius of Na^+^ and K^+^ resulting in the most rapid Li^+^ diffusion rate. Second, from the CV and galvanostatic current charge and discharge curves, it could be demonstrated that surface adsorption played an important role in the charge and discharge processes of SIBs (unlike LIBs and KIBs, which mainly involve a two-phase reaction), which may have led to the slower diffusion rate of Na^+^ in the bulk phase. It should be noted that the calculation was based on the value of the redox peaks of CV curves, which reflected the two-phase reaction during charge and discharge processes. Thus, the calculated value only presented the diffusion rate corresponding to the two-phase reaction, rather than the true diffusion rate of Na^+^. Therefore, the calculated diffusion rate of K^+^ was higher than that of Na^+^.

## 4. Conclusions

In summary, we synthesized Sb_2_S_3_@PPy coaxial nanorods as versatile anodes in LIBs, SIBs, and KIBs with excellent electrochemical performance. Specifically, it showed a reversible capacity of 940 mAh g^−1^ at 0.1 A g^−1^ (SIBs), higher than those of similar works. The Sb_2_S_3_@PPy coaxial nanorods also showed excellent rate capacity and cyclic stability in SIBs. Additionally, the reversible capacity of Sb_2_S_3_@PPy nanorods reached up to 700 mAh g^−1^ at 0.1 A g^−1^ (KIBs). A systematic study was carried out on the electrochemical behavior of Sb_2_S_3_@PPy nanorods in lithium, sodium, and potassium storage, including cycle performance, rate capacity, galvanostatic current charge and discharge, and CV curves under different scan rates. This study provides a universal model for the study of chalcogenide compounds as anodes for alkali metal ion batteries.

## Figures and Tables

**Table 1:** Comparison of Sb_2_S_3_ based anode materials for SIBs and KIBs in terms of their composition, reversible capacity, cycle life, and rate performance.

	SIBs			KIBs			Ref.
Electrode Material	Reversible Capacity (mAh g^−1^)	Cycle Life (mAh g^−1^)	Rate Performance (mAh g^−1^)	Reversible Capacity (mAh g^−1^)	Cycle Life (mAh g^−1^)	Rate Performance (mAh g^−1^)	
**Our work**	**940 @ 0.1 A g^−1^**	**881 @ 0.1 A g^−1^** **after 50 cycles**	**940@ 0.1 A g^−1^** **490 @ 1 A g^−1^**	**700 @ 0.1 A g^−1^**	**487 @ 0.1 A g^−1^** **after 18 cycles**	**690 @ 0.1 A g^−1^** **280 @ 1 A g^−1^**	
CNT@Sb_2_S_3_@PPy	596 @ 0.1 A g^−1^	500 @ 0.1 A g^−1^ after 80 cycles	596 @ 0.1 A g^−1^ 400 @ 1 A g^−1^	-	-	-	[30]
Sb_2_S_3_@PPy	600 @ 0.1 A g^−1^	427 @ 0.1 A g^−1^ after 50 cycles	600 @ 0.1 A g^−1^ 500 @ 1 A g^−1^	-	-	-	[31]
Sb_2_S_3_@C	700 @ 0.2 A g^−1^	650 @ 0.2 A g^−1^ after 50 cycles	700 @ 0.2 A g^−1^ 450 @ 0.8 A g^−1^	-	-	-	[32]
3D SbNPs@C	-	-	-	488 @ 0.2 A g^−1^	461 @ 0.2 A g^−1^ after 15 cycles	478 @ 0.2 A g^−1^ 288 @ 1 A g^−1^	[33]
Sb_2_S_3_-SNG	-	-	-	530 @ 0.1 A g^−1^	500 @ 0.1 A g^−1^ after 100cycles	-	[34]

**Table 2:** Diffusion coefficient of alkali metal ions of Sb_2_S_3_@PPy anode.

Alkali Metal Ions	Diffusion Coefficient (cm^2^ s^−1^)
Li^+^	3.16 × 10^−10^
Na^+^	7 × 10^−11^
K^+^	1.78 × 10^−10^
